# Art, auto-mechanics, and supramolecular chemistry. A merging of hobbies and career

**DOI:** 10.3762/bjoc.12.40

**Published:** 2016-02-26

**Authors:** Eric V Anslyn

**Affiliations:** 1 Department of Chemistry, University of Texas, Austin, TX 78712, USA

**Keywords:** art, assembly, complexity, function, mechanical, supramolecular

## Abstract

While the strict definition of supramolecular chemistry is “chemistry beyond the molecule”, meaning having a focus on non-covalent interactions, the field is primarily associated with the creation of synthetic receptors and self-assembly. For synthetic ease, the receptors and assemblies routinely possess a high degree of symmetry, which lends them an aspect of aesthetic beauty. Pictures of electron orbitals similarly can be seen as akin to works of art. This similarity was an early draw for me to the fields of supramolecular chemistry and molecular orbital theory, because I grew up in a household filled with art. In addition to art, my childhood was filled with repairing and constructing mechanical entities, such as internal combustion motors, where many components work together to achieve a function. Analogously, the field of supramolecular chemistry creates systems of high complexity that achieve functions or perform tasks. Therefore, in retrospect a career in supramolecular chemistry appears to be simply an extension of childhood hobbies involving art and auto-mechanics.

## Review

### Introduction

**Figure 1:**
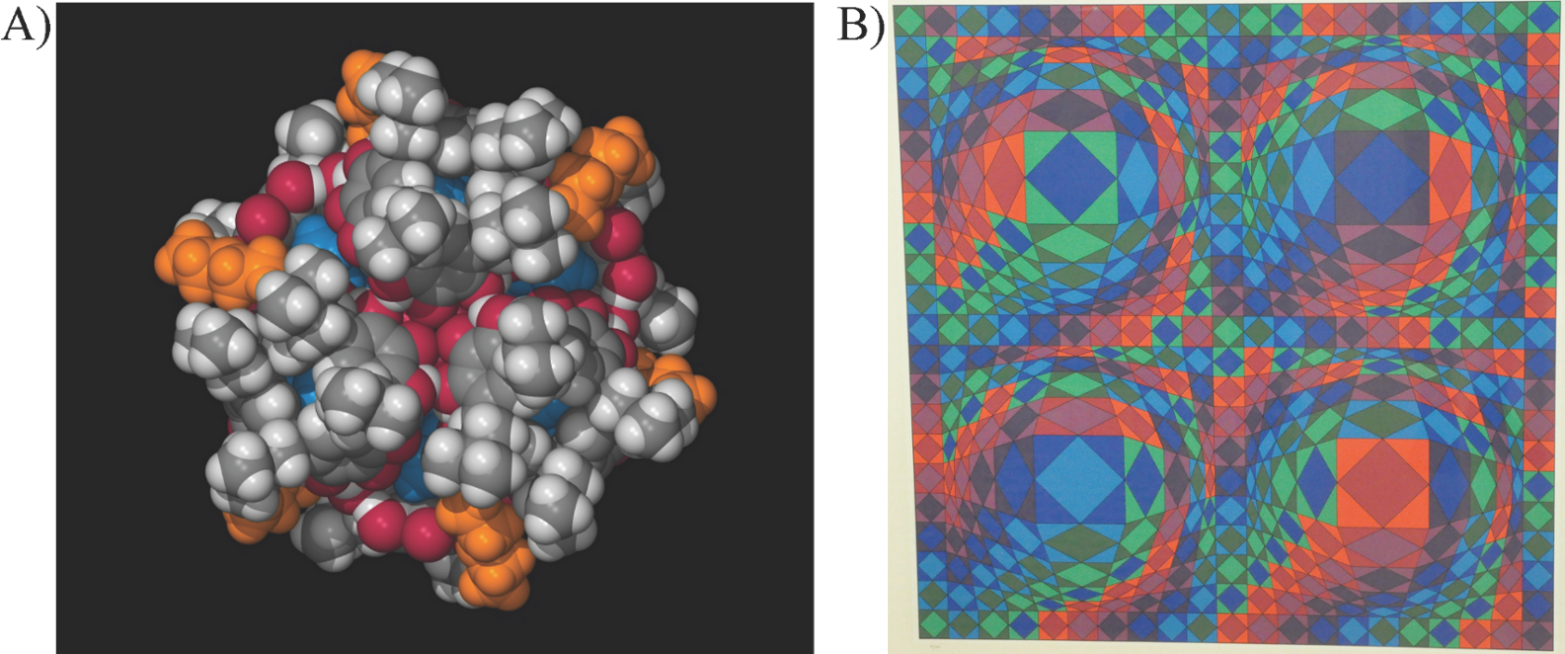
A) An Atwood Cluster, picture donated from Jerry Atwood. B) Vasarely serograph, personal photograph from EVA.

The field of supramolecular chemistry abounds with beautiful and aesthetically pleasing molecules. From Stoddart’s rotaxanes [1–2], Sauvage’s knots [3–4], Rebek’s capsules [5], Fujita’s 3-D MOFs [6–7], to Atwood’s clusters [8–9], our field is associated with creating complex structures, often of very high symmetry. This makes ChemDraw structures, space-filling models, or ball and stick renderings very akin to objects found in modern art [10]. Can one look at an Atwood cluster without thinking of Geometric Abstract Art? Maybe one can, but the similarly is striking (Figure 1).

Besides having an aspect of beauty, supramolecular structures are created to achieve a chemical function or task. These functions range from imparting mechanical changes [11–13], to altering material properties [14–15], to manipulating biological ramifications [16]. Thus, not only are the assembled chemical entities visually striking, they also have real-life practical applications. This combination of art and function undoubtedly had a large influence on why my career transitioned into the field of supramolecular chemistry.

### Earliest inspirations

**Figure 2:**
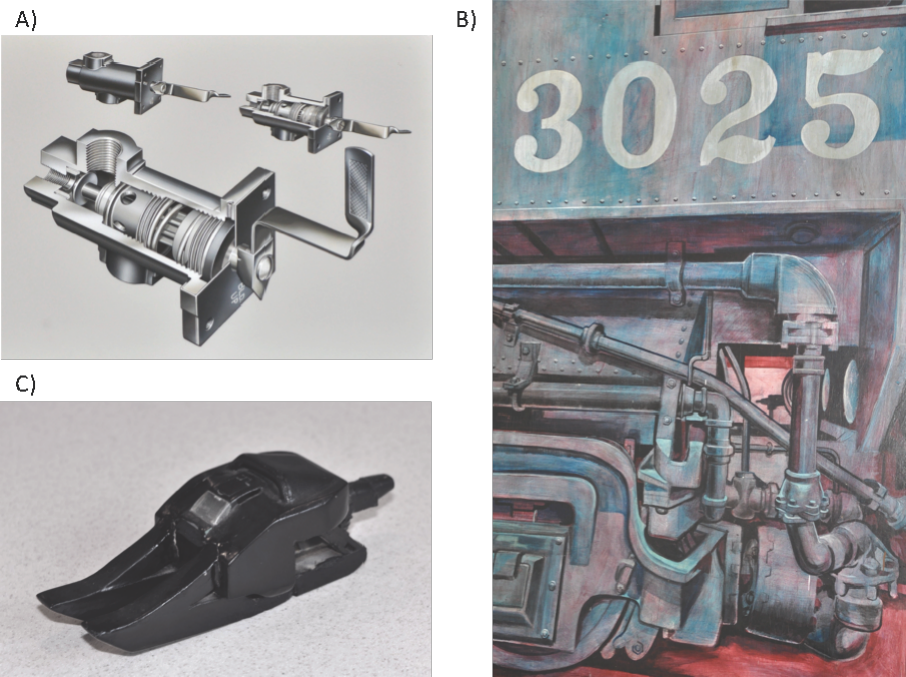
A) An airplane part air-brush rendering (S. S. Anslyn, 1950’s). B) A mural of a locomotive engine (S. S. Anslyn, 1971). C) A “destructo” created by Brian Heidsiek in approximately 1973. All graphics are personal photographs by EVA, who has the copyright to every photo used herein.

My father, Samuel Anslyn Jr., was an industrial artist. In World War II and later he worked as an artist rendering exquisitely detailed charcoal sketches, and airbrush mock-ups of airplane and ram-jet parts. Before becoming an art and drafting teacher at a Glendale Community College, he was the art director at Marquardt Corporation [17]. My house and garage are filled with the most wonderful renderings of airplane parts (Figure 2A), as well as large oil paintings of other industrial and mechanical structures, such a locomotives (Figure 2B). As he aged, his need to express creativity converted to being an auto mechanic, restoring old Jaguars and Porsches to the level of award winning Concours D’Elegance vehicles.

Growing up in a household with a father that was both an artist and a mechanic, it became natural to build plastic and balsawood models as a hobby and to work on motorized vehicles. My neighbor from age 7, Brian Heidsiek, became an industrial designer himself. As children we raced and worked on go-karts. We also created numerous models from scratch, culminating in what were known as a series of “destructos”, i.e., vehicles that were indestructible and saved the world from disasters (Figure 2C), inspired by the cartoon show “The Thunderbirds” [18]. Even to this day, my hobbies still involve go-kart racing and restoring old cars.

**Figure 3:**
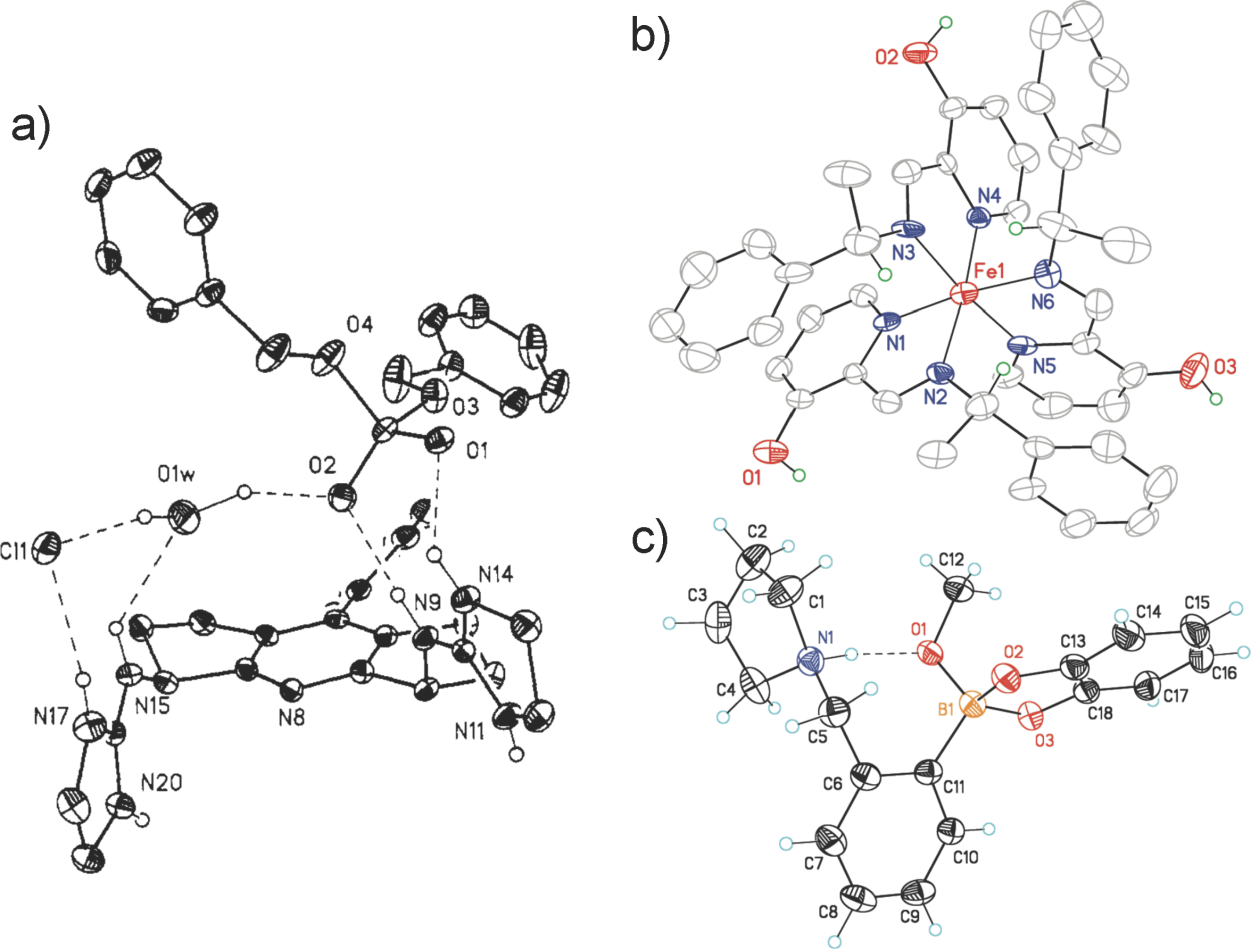
Representative crystal structures of various complexes we have created over the years, that in my own opinion are particularly beautiful. Collage reproduced with permission from renderings in reference [19–21]. Copyright 1993, 2009, and 2012 The American Chemical Society.

Thus, after 40 years of hindsight – considering my childhood with an artist/mechanic for a father, and a best friend with whom I built functional models, it is not surprising that my career has focused on the creation of new molecular and supramolecular structures designed to execute a particular function or achieve a certain task. Further, while we all know “beauty is in the eye of the beholder”, many of our group’s chemical structures are exquisite, at least in my own somewhat biased opinion. In fact, even after 27 years as a Professor, I experience a thrill when we get a crystal structure because they invoke an aesthetic response (Figure 3). The combination of art and function is fully analogous to both my father’s and neighbor’s designs, except that the “art” of my group is visualized on the nanoscopic scale rather than on a macroscopic scale of my dad and friend. Thus, my childhood exposure to the combination of art and function has clearly led me to the field of supramolecular chemistry.

### Origin of a love of organic chemistry, orbitals, and complexity

All organic chemists, and in particular supramolecular chemists, must share an enjoyment in creating new chemical entities of our own inspiration. My passion for organic and organometallic synthesis was first developed when performing undergraduate research at the California State University Northridge (CSUN) under the tutelage of Dr. Edward Rosenberg. At this undergraduate institution my major was pre-med, with all the associated drive and motivation to do well, accompanied with the annoying behavior of such students. For example, my major was chemistry solely because a larger fraction of B.S. chemistry majors were accepted to medical school than other majors. I had never taken a chemistry class in high school, yet it was my declared major. Further, because the counselors advised that undergraduate research was a good exercise to build a curriculum vitae for entrance into medical school, research was one of my pursuits from freshman year throughout my undergraduate career. The project entailed the use of variable-temperature NMR to measure the dynamics of ligand migration in trimetallic osmium clusters [22–23]. Dr. Rosenberg was an inspirational figure, and his pursuit for scientific knowledge was infectious. In particular, he imparted a love of deciphering mechanistic puzzles.

To me, mechanistic puzzles are similar to deciphering how to fix an internal combustion engine; both involving diagnosis of the problem within a large “black box” and fixing it with tools appropriate for the job: valve spring compressors, feeler gauges, socket wrenches, etc. As organic chemists, we propose hypotheses explaining how Mother Nature works, and we have particular experimental tools to test our theories: kinetics, isotope effects, solvent effects, etc. [24].

**Figure 4:**
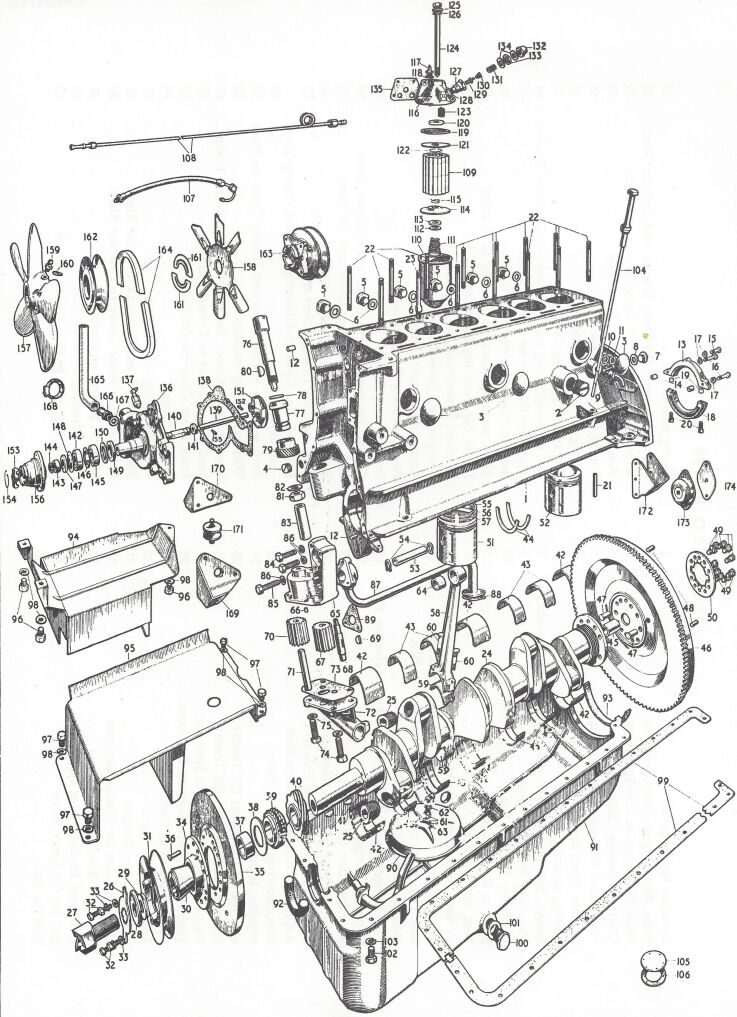
Exploded view of a 1953 Mk VII Jaguar in-line six internal combustion motor (bottom end), overhauled by EVA in 1976. Personal photograph by EVA.

Reactions with multiple components all working in concert to achieve a function beyond that of the individual parts (known recently as “emergent properties” [25–26]) is likewise analogous to an internal combustion motor (Figure 4). Numerous parts: pistons, values, crankshafts, cams, etc., all combine together to create a force that propels the automobile. The expanded version of a 1953 in-line six-cylinder bottom-end from an Mk VII Jaguar owner’s manual is a powerful image that accentuates the idea of emergent properties.

As described below, the field of differential sensing, in which our group works extensively, takes the responses from a suite of receptors and creates patterns for diagnostic purposes that are beyond what can be achieved by the separate components alone. In fact, we are currently working to push this even further creating multicomponent cascades of reactions yielding a final result that the individual reactions themselves cannot achieve. With the hindsight described in this article, it is clear that my inspiration to pursue such research is driven by similar hobbies from my childhood.

**Figure 5:**
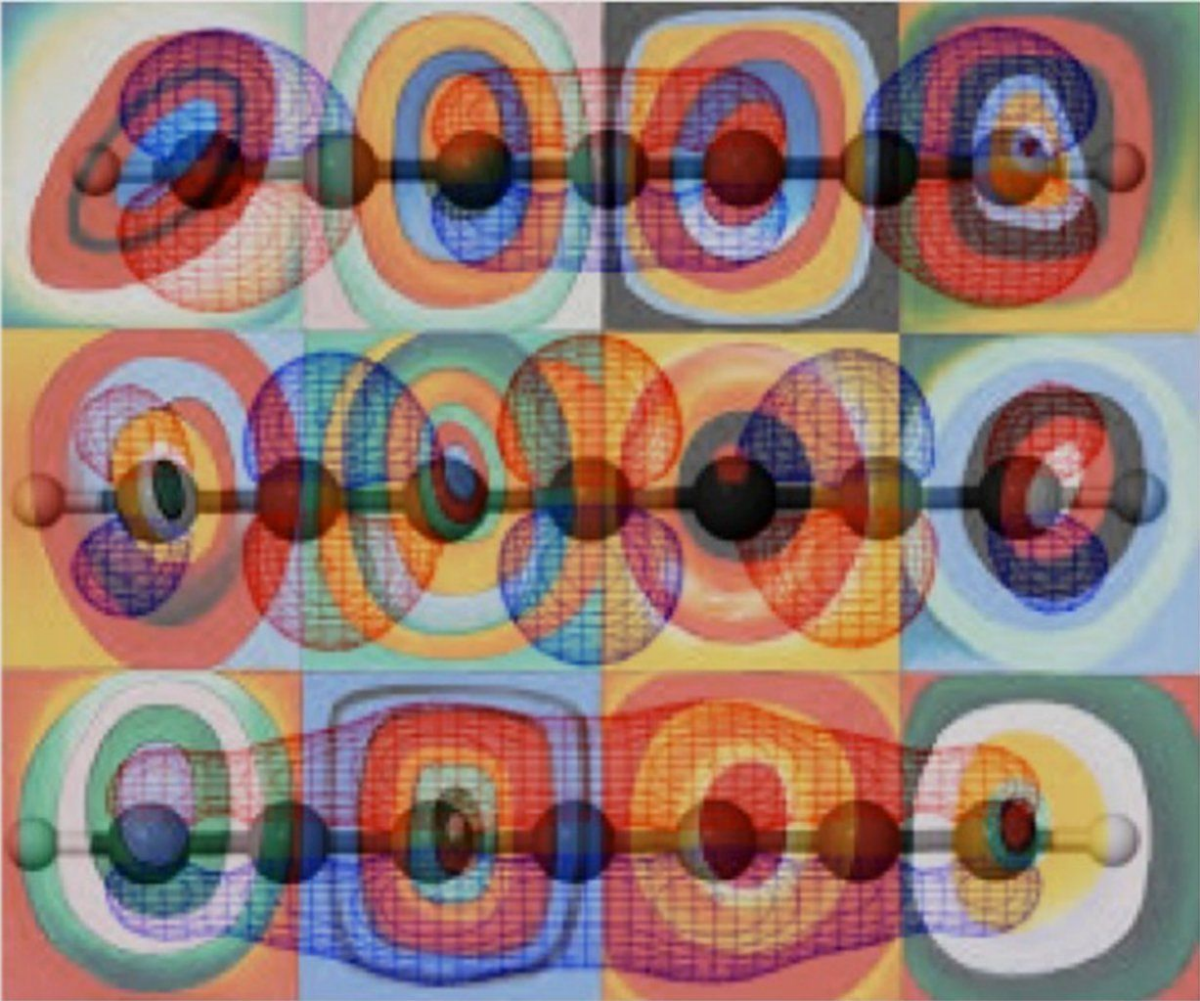
Kandinsky’s Concentric Circles. (http://amazinglittleartiststves.weebly.com/student-artwork/category/kandinsky) , with orbitals computed by John Stanton (personal communication). Collage created by EVA.

From my first introductory organic chemistry class I have had a fascination with electron orbitals. The artistic similarity and aesthetic reaction to molecular orbital theory is obvious. Even if beauty is in the eye of the beholder – can anyone really question that HOMOs and LUMOs (Figure 5) are beautiful representations? When considered in this manner, electronic structure theory takes on a completely different aspect, that Kandinsky would have appreciated [27].

### Graduate school and Post-Doc

After receiving a B.S. in chemistry from CSUN, medical school at the University of Southern California (USC) was the next destination. But, this lasted only about two weeks. In the evenings, my inorganic and organic chemistry textbooks [28–29] were calling to me rather than required physiology and anatomy books. Thus, after withdrawing from USC, the next year was spent continuing research with Ed Rosenberg and creating signs. With my friend Andy Trapani, we started “Eric’s Signs”. This company made Styrofoam lettering for sides of buildings modeled off of a company’s business card and logo. It was quite successful and could have blossomed into a business career in industrial art.

After a one-year break from schooling, Caltech was my destination, where Dr. Robert Grubbs accepted me into his group. This was one of the most important and impactful decisions of my life. Dr. Grubbs was another inspirational individual with a love of science that permeated through his group. He has an innate instinct of when chemistry will and will not work. Part of my Ph.D. thesis was computational under the direction of Dr. William Goddard, involving molecular orbital theory and the rendering of orbitals. Both Professors taught me various aspects of the art of physical organic chemistry. In addition, Dr. Dennis Dougherty welcomed me to his group meetings, where various topics of supramolecular chemistry were common. Little did either of us suspect we’d co-author a physical organic textbook together about 15 years later [24].

Using the combined experience from Grubbs, Goddard, and Dougherty, physical organic chemistry as applied to biological problems was a main interest, which became the topic of my post-doctoral work with Dr. Ronald Breslow at Columbia University. Breslow has the quickest mind of anyone I’ve ever met and his enthusiasm for his group’s work knows no bounds. When he would enter a laboratory to hear the latest news, it was an explosion of energy. He wanted to hear about everything, even the latest TLC conditions. The atmosphere of his group inspires all members to go as far as possible in academia.

### Early academia

**Figure 6:**
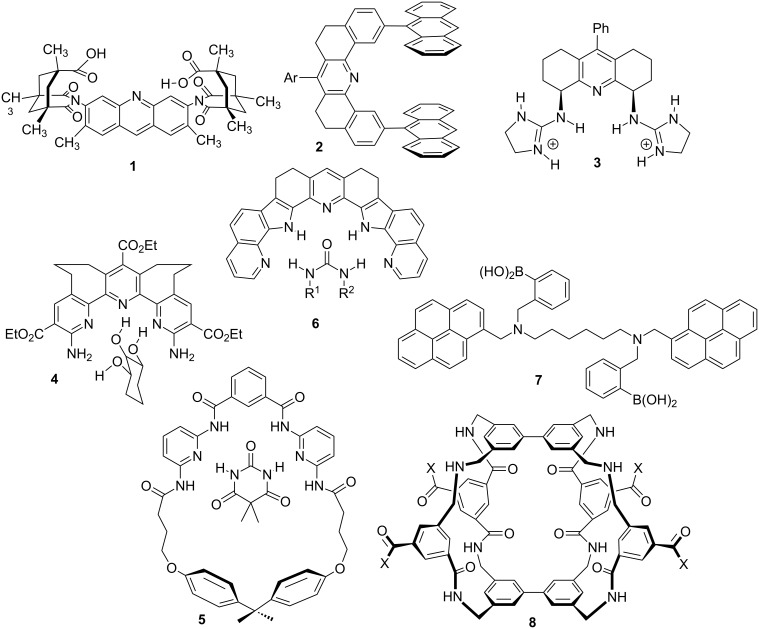
A potpourri of chemical receptor designs that influenced our group’s work **1**, **2**, **5**, **6**, **7**, **8**), along with a few of our own (**3** and **4**) [34–45].

The question everyone has to confront when starting in academia is “what to do?”. Initially I took an easy route. My post-doctoral work with Ronald Breslow was focused on enzyme mimics for the hydrolysis of RNA [30–31]. Thus, continuing in this vein but using a different approach, that of guanidinium groups in preorganized scaffolds that created clefts, was the route my group pursued [19,32–33]. This was the era in supramolecular chemistry of Rebek’s Kemp-triacid clefts (**1**) [34–35] and Zimmerman’s tweezers (**2**) [36–38]. My own molecular designs were also reminiscent of these precedents (**3**) (Figure 6).

From 1989–1993 achieving tenure was a major goal, and I believed pursuing novel biologically relevant targets using supramolecular chemistry would make a unique and new contribution. Thus, our group created some of the earliest reported monosaccharide receptors (**4**) that exploited hydrogen bonding based recognition in chloroform [39–40]. The receptor designs from Hamilton (**5**) [41] and Thummel (**6**) [42] at around the same period of time clearly influenced my own designs. This was approximately 1992, and looking back at the polyol receptors (such as **3**) shows how far this field progressed. The use of boronic acids of Shinkai/James (**7**) [43–45] and the large cavities reported by Davis (**8**) [46] for binding saccharides has advanced the field far beyond our primitive designs (Figure 6).

The synthetic receptors we created were designed to answer basic science questions about enzyme mechanisms [19,47], rate enhancements from ion-pairing and general acid-catalysis [48], as well as reveal the strengths of hydrogen bonding. Interspersed among this work using synthetic receptors there was a continued fascination with approaching mechanistic problems using more classical physical organic methods, and we published a series of papers on the mechanisms of glycoside [49–50] and phosphoester hydrolysis [51].

### Why textbooks?

After achieving tenure, it is natural to reflect “Whew, what now?” In answering such questions, one dominant thought continued to recur – the most influential people on my perception and knowledge of chemistry were Grubbs, Breslow, Rosenberg, Lowry and Richardson, as well as Morrison and Boyd. These later two set of individuals were the authors of my graduate [52] and undergraduate [29] textbooks on organic chemistry, respectively. This thought brought the realization that a textbook has a far broader and extended effect on influencing how students think about chemistry when compared to what our research was ever likely to achieve. Thus, through a series of fortunate events the graduate-level textbook “Modern Physical Organic Chemistry”, co-authored with Dennis Dougherty, was the result [24]. Similarly, due to a long friendship with Brent Iverson, dating back to graduate school, the undergraduate book “Organic Chemistry” was produced [53]. Writing the graduate level textbook was the most educational thing I’ve done, and ranks among the most gratifying experiences of my career.

### After tenure

Much of our group’s work prior to tenure was addressing mechanistic aspects on the timing of proton transfers in hydrolysis reactions [51,54–56]. While the approaches we used to answer these questions where mechanistically interesting, there was a question – “How many people really care about such subtle details?” Thus, while mechanistic pursuits remain a constant in our group’s work, we switched from using synthetic receptors as mechanistic probes to using such receptors for sensing purposes. The impetus for doing so was driven by an attempt to have a higher impact with our work, but, admittedly, was also due to serendipity.

Several events moved our group’s research toward sensing applications. One was having a synthesis to create **9** as an RNA hydrolysis catalyst. Structure **10** was building up in a vial as a byproduct of synthesizing **9**, and we had no idea what to do with it. That was until one day sitting at my desk drinking a Fresca soda and reading the ingredients, the first of which was sodium citrate, the light bulb went off - Bingo! Compound **10** should definitely bind citrate quite strongly, even in a highly competitive media, due to the fact that all the hydrogen bonding will be strengthened from the additional ion-pairing (**10**). This hypothesis proved to be correct [57]. Hence, the idea was to use **10** as an optical sensor for citrate. But, we needed a signaling protocol, and neither **10** nor citrate possess a chromophore. The usual approach would have been to covalently attach a chromophore to the receptor, but we wanted a more general approach, one that would not require additional synthesis. Upon remembering that the Breslow group would follow the displacement of fluorophores bound in the cavity of cyclodextrins to measure *K*_eq_ values, our idea was to instead exploit the displacement as the sensing modality. Thus, the idea of an indicator-displacement assay (IDA) was born [58–59]. As with so many “new” ideas in chemistry, the approach had actually been used before, by Inouye and Shinkai [60–61]. IDAs are now one of a handful of standard approaches to creating optical sensors [62].

**Figure 7:**
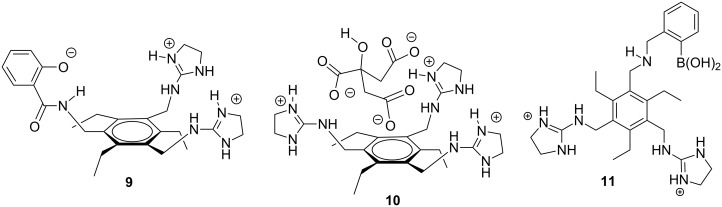
Evolution of design of our citrate receptor [63–67].

Our group optimized the citrate receptor design by incorporating a single boronic acid (**11**) [63] and measured citrate in soda pops [64], vodkas [65], and most recently showed that such receptors can be used in dialysis clinics to monitor citrate anticoagulation therapy [66]. The optimization procedure for the citrate receptor followed a classic “lock and key” design strategy (Figure 7). The receptor was preorganized by the hexasubstituted benzene [67] to present two guanidinium groups and the boronic acid in a spatial manner to best complement the citrate “key” to the receptor “lock” (**11**). In other studies, using a lock and key design approach led to very selective and high affinity receptors for heparin [68–69] and 2,3-bisphosphoglycerate [70].

Although reading the label of a Fresca soda indeed sparked the idea of pursuing a citrate sensor, the idea of working on sensing had been percolating in my mind for a while. A. P. De Silva was pioneering the use of PET (photoinduced electron transfer) signaling [71], Seiji Shinkai (and his post-doctoral associate Tony James) were creating sugar sensors [43–45], and Anthony Czarnik had published his landmark treatise “Desperately Seeking Sensors” [72]. These three individuals are the true fathers of “Supramolecular Analytical Chemistry”, even if our group later introduced this terminology [73].

### Sensing a paradigm shift

Whereas selectivity has been a goal for many studies using synthetic receptors, in part because Clark Still once noted that synthetic receptors could be as selective as antibodies [74], a chance lunch led me to consider moving in an entirely different direction toward the end of the 20^th^ century. My ex-colleague, Dr. John McDevitt (now at NYU) told me over lunch about devices known as “electronic noses” [75–76], which are analytical devices that have arrays of cross-reactive entities whose signals (most commonly electrochemical) can be deciphered by chemometric routines to create patterns (also called “fingerprints”) for the composition of gases/vapors. In collaboration with McDevitt, as well as Drs. Jason Shear and Dean Neikirk, we created one of the earliest “electronic tongues”, which simply meant we were analyzing solution compositions rather than gases [77]. Our device emulated design principles emanating at that time from David Walt [78–79] and Ken Suslick [80–81], who were pursuing similar goals.

The field of electronic noses and tongues has a biomimetic origin. Having worked with Breslow as a post-doctoral fellow, who coined the word biomimetic [82], the idea of mimicking the mammalian senses of taste and smell as an approach to chemical sensing seemed obvious. Mammalian chemical sensors do not use highly selective, lock and key-like, receptors, but instead rely on a series of low-selectivity but cross-reactive receptors that create a pattern [83]. These patterns act as fingerprints, to recognize and diagnose future foods and beverages. Whereas the field of electronic noses in the late 1990s was very sophisticated, in general the analytical chemistry community did not incorporate principles of supramolecular chemistry into their designs, and furthermore were primarily limited to vapor analysis.

We did not have an immediate epiphany that the supramolecular community could have a large impact in this field, but instead this realization came gradually, as studies from the group led to the conclusion that a lack of selectivity could be powerful. Furthermore, it was a reviewer of one of my early grants in this area that made me realize what I had not already recognized; we were, and still are, “making lemonade out of lemons”. In essence, we were taking advantage of the fact that synthetic receptors lack a high level of selectivity.

One of the earliest studies from our group that revealed how a lack of selectivity could be useful involved the age of scotch whisky [84]. We found that the same receptor we had optimized for citrate (**11**) would indiscriminately bind tannic acids, which are species that leach from oak barrels as the whisky ages. Using this one receptor to signal all tannic acids, we could create an IDA that correlated with the age of the whisky. In a second similar study, we took advantage of our early fledgling interest in chemometrics (see more below), and showed how an artificial neural network (ANN) could be used to analyze mixtures of cross-reactive receptors with indicators to accurately quantitate concentrations of the very similar analytes malate and tartrate [85]. Subsequently, the Severin group has also nicely exploited both mixtures of receptors and indicators [86–87], as well as spatially arrayed versions, to diagnose other very subtle differences in analytes [88].

To distinguish the idea of using selective receptors from that of using cross-reactive arrays we coined the term “differential sensing” [89]. The idea was to highlight the most important factor in this biomimetic approach – that the receptors all acted differently from one another. The responses from all the receptors would need to be interpreted by a chemometric protocol [90], such as principal component analysis (PCA), linear discriminate analysis (LDA), hierarchical cluster theory (HCT), or an ANN. A course at Georgia Tech University, held in approximately the year 2000, was my basic training in the methods. Admittedly, the extensive linear algebra discussed was above my comprehension, but much of what was taught ultimately resulted in a manuscript that we hope helps the supramolecular chemistry community to use these methods [91].

**Figure 8:**
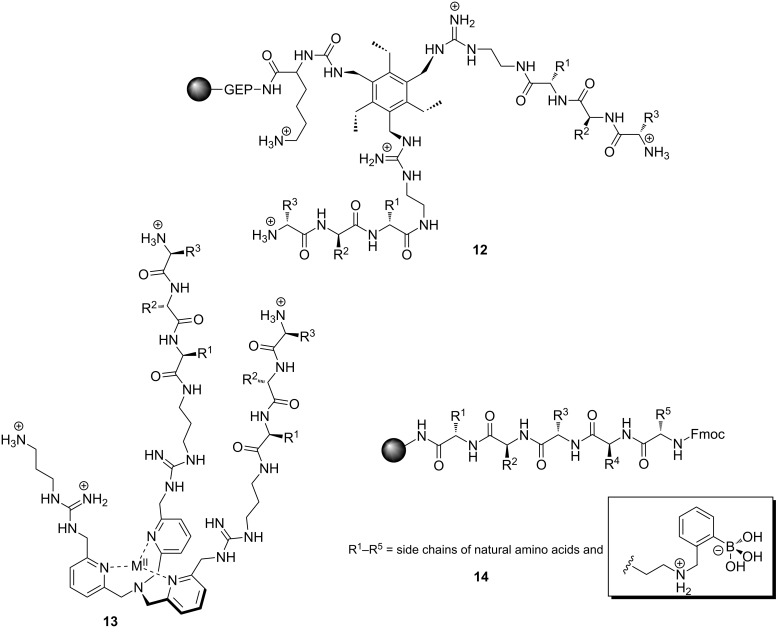
Combinatorial peptide library designs used for differential sensing purposes [92–94].

Among the earliest work from our group using the electronic tongue and chemometrics were methods to differentiate ATP, GTP and AMP [92], phosphorylated peptides [93], as well as a technique to identify sweeteners in coffee and tea [94]. Each of these studies used combinatorial libraries of peptides around a preorganized scaffold or with a known targeting agent; these include **12**, **13**, and **14**, respectively (Figure 8). Such achievements would have been difficult for supramolecular chemistry groups using the standard lock and key approach to create synthetic receptors due to the difficulties in creating receptors with the appropriate specificity.

In the beginning, the supramolecular chemistry community was not receptive to this kind of work. Quite well known chemists, and close friends, had comments such as “you’re going to put us all out of business”, “this is not science”, or “you’ve lost your way”.

**Figure 9:**
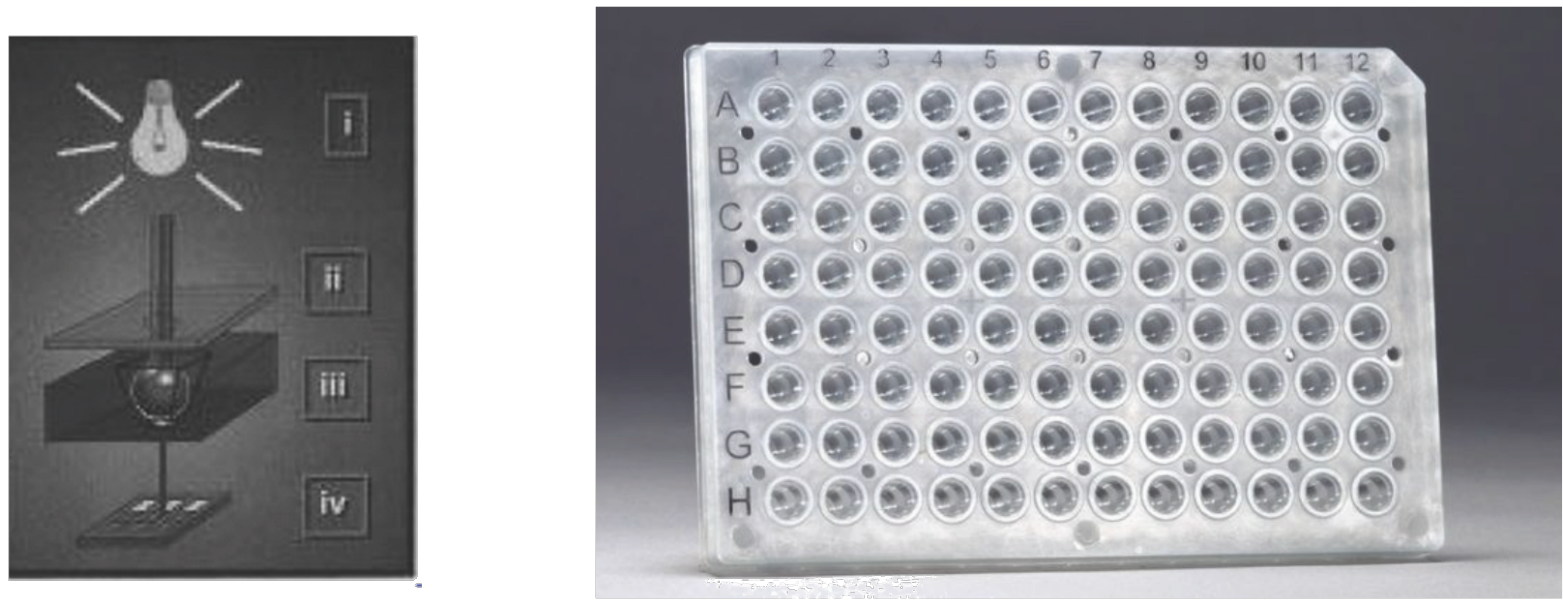
Concept behind the electronic tongue, with micromachined divets that hold beads placed in an array. While not micromachined, a much simpler analog that accomplishes much of the same concept is just a simple 96-well plate.

The electronic tongue created with McDevitt possessed beads placed in micromachined divots on a silicon chip (Figure 9A), and solvents and samples were introduced to the system via an external HPLC [69]. While the miniature nature of the system was intriguing, such a device was going to be difficult for supramolecular chemists to adopt. More user-friendly variants for spatially arraying receptors for solution analysis were thus created by others, such as Pavel Anzenbacher’s sol–gel approach [95–96]. Yet, the absolute easiest way to array receptors is to use commercially available plates, such as plastic 96-well plates, because the receptors can be dissolved and then dispensed by common 8- and 12-channel pipettes. To our knowledge, one of the earliest reports of using such a plate with supramolecular chemistry and chemometrics sensing techniques was from Lavigne [97–99]. His work inspired our own group to move to 96-well plates, and from that point forward the field of differential sensing has exploded (Figure 9B) [100]. But, for my own tastes the work from Vince Rotello on biological applications [101–102] should inspire supramolecular chemists to move the field toward pathology.

**Figure 10:**
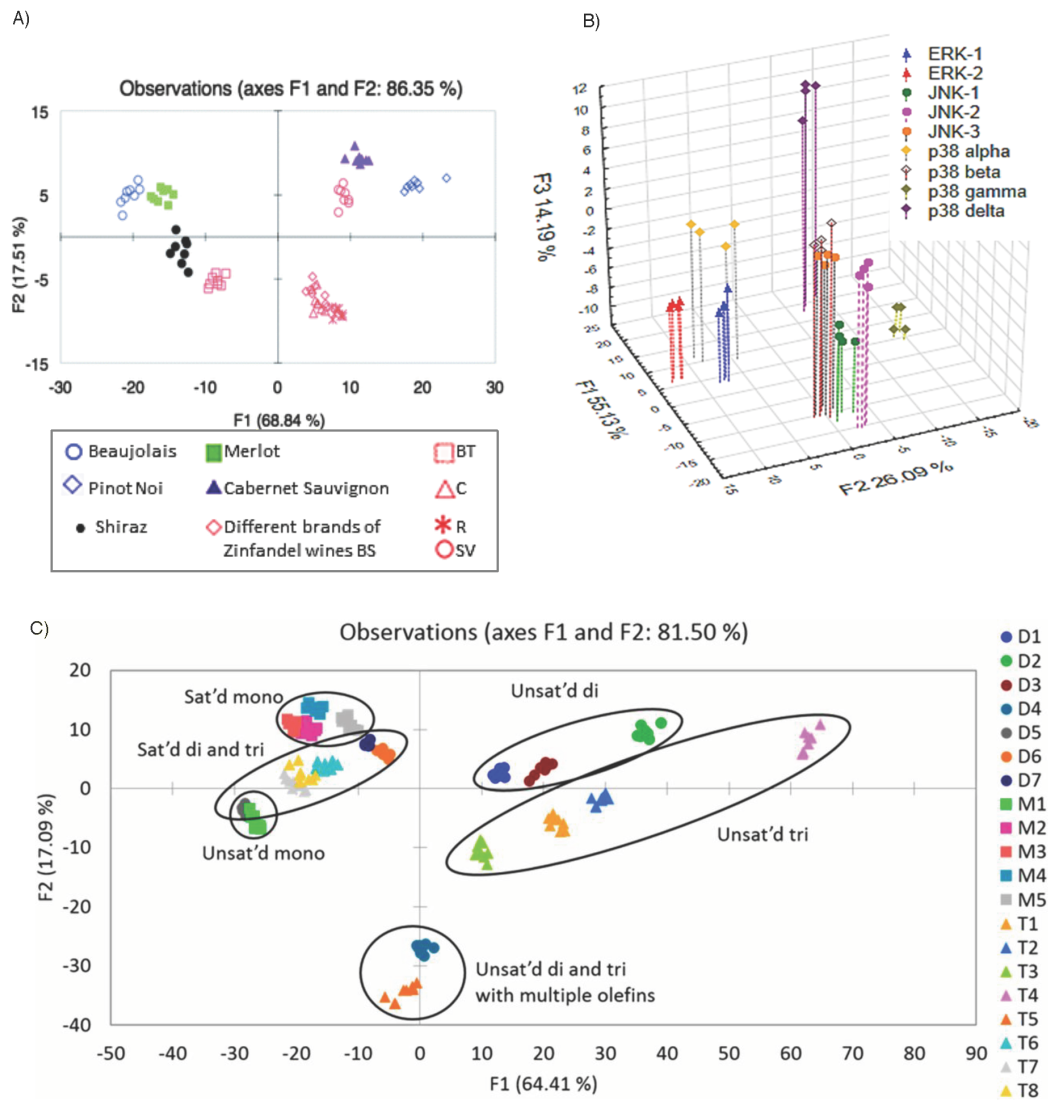
a) LDA plot of the response from different wine varietals with array Z [103]. b) Three-dimensional LDA plot of the response from the SOX-peptides showing in vitro differentiation of nine MAP kinases [106]. c) LDA plot of data collected from 96-well plates [107]. The array components consisted of BSA and HSA (100 µM), glyceride (90 µM), DNSA (60 µM), ANS (60 µM), NBD-FA (60 µM), metathesized glyceride (90 µM), AF (100 µM), and DNSA (60 µM) in phosphate buffer with <5% (v/v) THF. Cross-validation: 98%.

In our own laboratories the most recent uses of differential sensing have focused upon trying to push the limits of the technique. Three studies were driven by attempting to see how far the idea of using cross-reactive arrays could be pushed for solution-based analysis of complex, and/or subtly different, analytes. Probably the most well-known set of studies from our group are on wines. As with our earlier work, the approach uses a suite of combinatorial peptides as differential receptors. The peptides are biased with a large fraction of the amino acid histidine, are metallated with Cu, Ni, and Zn, and bind indicators to create a series of IDAs that can classify wine varietals, hang time, correlate with the human taste response of astringency (Figure 10A), and identify percentages in blends [103–105]. While this work was started simply as a means of seeing if we could create assays that would parallel human taste responses, we have since found that wine fraud may be a real-life application for the method.

As has Rotello, we are taking our differential sensing work to the biological arena. Kinases are enzymes that are involved in cellular signaling and regulation. Monitoring their activity has commonly involved the creation of highly selective peptides that respond to only one kinase [108–109]. This can be viewed as a lock and key approach, while as described herein, many groups have shown that the differential sensing approach may be more applicable for certain applications. Thus, we took a suite of peptides containing the SOX fluorophore, and analyzed their ability to classify MAP kinase identity, concentrations, and inhibitors thereof [106,110]. The chemometric analysis of the data (Figure 10B) revealed that most of the peptides were phosphorylated by each kinase, and that unexpected activity was found for inhibitors.

**Figure 11:**
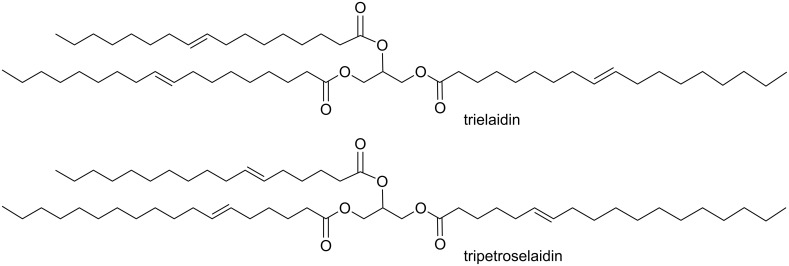
Two seemingly impossible targets to make highly selective receptors for.

Among the most challenging guests that we could envision for supramolecular chemistry to tackle are glycerides. Glycerides often differ only by numbers of methylene groups, positions of double bonds, and stereochemistry of the olefins. It seems impossibly difficult to create a synthetic receptor that could bind selectively trielaidin over trielroselaidin (differing only by double bond position in each fatty acid chain, Figure 11) and a second synthetic receptor that did the opposite. Thus, if successful, the demonstration of a differential sensing approach that could classify glycerides, determine their structural features, and quantitate concentrations, would be a large validation of the method. To accomplish this we used serum albumins as the cross-reactive receptors, paired with a series of hydrophobic indicators [107]. The method worked extremely well (Figure 10C), and we are currently pursuing the analysis of adipocyte extracts in collaboration with Sanofi–Aventis for diabetes studies.

### What’s next?

Where should the field of supramolecular analytical chemistry be moving, and therefore what inspirations are there for our group? Undoubtedly, because it is my first love, my group will continue to study mechanisms of organic reactions, molecular recognition, and photophysical techniques. In each study, we’ll be driven to create imaginative new approaches and complex physical entities of beauty that perform functions and tasks. As discussed above, we’ll continue to create differential sensing arrays for new, ever-expanding applications. However, after the analysis of glycerides, we feel there is no longer a need to see how challenging a class of analytes can be tackled. Instead, it is now necessary to make the methods truly practical for real-life applications.

But, far more important than my own group’s work, to survive and thrive, supramolecular analytical chemistry must create results that are widely recognized by the chemical community. Our field has to have broad impact, not only advancing the basic science of molecular recognition and chemical reactivity, but also using this information to influence how other scientists perform their own studies. In this regard, differential sensing must find a “Grand Challenge” that it, and only it, can solve. The first artificial nose companies, Aromascan and Cyrano, have fallen by the wayside, primarily for lack of a real market application. It is true that solution-based differential sensing is likewise struggling for a solid foothold in the economy. In contrast, imaging agents are clearly a frontier. These are the kinds of sensors that Tony Czarnik originally envisioned [72], and commercial success has been achieved with the company Life Technologies, formally Molecular Probes.

The directions that Vincent Rotello is taking the field of differential sensing, toward that of biological applications, is one clear future. We are similarly moving to biological applications with kinase and lipid analysis, and cellular classification. But, even beverage analysis, complex mixture authentication, and drug metabolism, are still important areas for differential sensing applications.

In addition, the work of Scott Phillips [111–113] and Doron Shabat [114–116] are currently inspirational for our group’s research efforts. They are both using auto-inductive and cascade reactions, for signal amplification purposes. Given their advances, we are currently using our own physical organic chemistry insights to amplify the responses of molecular recognition in single analyte or array sensing. This is an area where supramolecular and physical organic chemists can create ensembles with many components that create properties that emerge which are greater than the individual parts alone.

## Conclusion

In summary, it is clear after a 27-year career in supramolecular chemistry that my group’s work is just a continuation of my childhood. This childhood was driven to emulate my father and have fun with my neighbor Brian. Just as I did during these formative years, my current group strives to make complex systems, with numerous moving parts, to achieve a function. This is similar to creating and fixing internal combustion motors on cars and go-karts, as well as designing and constructing from scratch balsa wood models. My current weekend hobbies are not any different, leading my wife to often observe “you’re replicating your childhood“. Further, while not necessarily designed to be objects of art, the compounds our group creates, and those that the field of supramolecular chemistry generally creates, are indeed beautiful. Even molecular orbital theory creates objects of worthy of artistic notice. This is what initially drew me to the field, and the aesthetic feelings evoked to the complexity of chemical assemblies are still my driving force for the creation of imaginative and novel systems chemistry.

If there is a lesson here, it is that one should take advantage of their strengths. Our hobbies as children, and as adults, don’t necessarily need to be significantly different than our careers. They can meld together, and thus work and recreation become one and the same.
